# Biodegradation of Phenanthrene by *Mycobacterium* sp. TJFP1: Genetic Basis and Environmental Validation

**DOI:** 10.3390/microorganisms13051171

**Published:** 2025-05-21

**Authors:** Shuyun Li, Jiazhen Liu, Ping Fang

**Affiliations:** College of Environmental Science and Engineering, Tongji University, Shanghai 200092, China; 2232769@tongji.edu.cn (S.L.); 2331349@tongji.edu.cn (J.L.)

**Keywords:** PAHs, *Mycobacterium*, phenanthrene degradation, bioremediation

## Abstract

The development of efficient bioremediation technologies for polycyclic aromatic hydrocarbons contamination is a hot research topic in the environmental field. In this study, we found that the *Mycobacterium* sp., TJFP1, has the function of degrading low molecular weight PAHs, and further investigated its degradation characteristics using the PAH model compound phenanthrene as a target pollutant. The optimal growth and degradation conditions were determined by single-factor experiments to be 37 °C, pH 9.0, and an initial concentration of 100 mg/L phenanthrene. Under this condition, the degradation efficiency of phenanthrene reached 100% after 106 h of incubation, and the average degradation rate could reach 24.48 mg/L/day. Combined with whole genome sequencing analysis, it was revealed that its genome carries a more complete phenanthrene degradation pathway, including functional gene clusters related to the metabolism of PAHs, such as phd and nid. Meanwhile, intermediates such as phthalic acid were detected; it was determined that TJFP1 metabolizes phenanthrene via the phthalic acid pathway. Simulated contaminated soil experiments were also conducted, and the results showed that the removal rate of phenanthrene from the soil after 20 days of inoculation with the bacterial strain was about 3.7 times higher than that of the control group (natural remediation). At the same time from the soil physical and chemical properties and soil microbial community structure of two levels to explore the changes in different means of remediation, indicating that it can be successfully colonized in the soil, and as a dominant group of bacteria to play the function of remediation, verifying the environmental remediation function of the strains, for the actual inter-soil remediation to provide theoretical evidence. This study provides efficient strain resources for the bioremediation of PAH contamination.

## 1. Introduction

Polycyclic Aromatic Hydrocarbons (PAHs) are typical Persistent Organic Pollutants (POPs) that widely distribute in soil, atmospheric, aquatic, and sedimentary systems [1,2,3,4,5]. These compounds exert toxicity through both direct biological effects and ecosystem disruption [6]. Furthermore, their bioaccumulation potential and triple genotoxic effects (mutagenicity, teratogenicity, and carcinogenicity) pose significant ecological and human health risks [7,8,9]. Since the United States Environmental Protection Agency (U.S. EPA) first designated 16 priority PAHs in 1979 [10], these compounds have been globally recognized as critical environmental contaminants. China has similarly designated PAHs as priority pollutants for environmental monitoring [11]. Consequently, developing safe and effective remediation strategies for PAH-contaminated matrices has become a major research priority in environmental sciences [9].

The remediation technologies in environments contaminated with PAHs mainly include physical, chemical, and biological methods [12,13,14]. Nevertheless, physical and chemical approaches typically entail high energy consumption, substantial costs, and potential secondary pollution, which limit their applicability for large-scale remediation [13,15]. In contrast, bioremediation, especially microbial degradation remediation, is more environmentally friendly and economical as it reduces the activity of harmful substances in the soil or breaks them down to harmless substances by utilizing the metabolism of microorganisms with specific functions under suitable environmental conditions [16,17].

Bioremediation leverages microbial enzymatic pathways to detoxify PAHs through two strategic approaches: bioaugmentation with exogenous degraders [18] and biostimulation of indigenous microbiota [15].

At present, microbial remediation technology is quite developed. Bioaugmentation technology, which means the introduction of functional strains or colonies of bacteria at contaminated sites, is the backbone of microbial remediation technology. And it has become a topical issue in recent years to study the remedy of PAHs pollution, while the screening of efficient degrading microorganisms has been the research focus of microbial enhanced remediation technology [19]. Bacteria are the most commonly reported microorganisms in PAHs degradation studies [20], with extensive evidence from diverse genera such as *Pseudomonas*, *Mycobacterium,* and *Sphingomonas* [18,21,22,23,24]. According to Huang et al. [25], 95% of the degrading bacteria belonged to the phylum of *Proteobacteria* and *Actinobacteria*, of which the most abundant genera were *Pseudomonas* (14%), *Rhodococcus* (10%), *Mycobacterium* (9%), and *Sphingobium* (7%).

Among these bacteria, *Mycobacterium* spp. demonstrated remarkable degradation efficiency and environmental adaptability. Specifically, *Mycobacterium* sp. SNP11 effectively degraded both phenanthrene and pyrene [26], while *Mycobacterium* sp. 6PY1 exhibited specific pyrene degradation capabilities [27]. Notably, *Mycobacterium* vanbaalenii PYR-1 displayed the broadest substrate range, capable of degrading naphthalene, phenanthrene, and pyrene [28]. *Mycobacterium* vanbaalenii PYR-1 is also the earliest *Mycobacterium* species isolated that can degrade PAHs and is one of the most widely and intensively studied strains in the field of prokaryotic degradation of high molecular weight PAHs. *Mycobacterium* spp. can be investigated in the field of polycyclic aromatic hydrocarbon degradation from a wide range of perspectives. *Mycobacterium* has not only shown efficient degradation of various PAHs in laboratory studies, but also has the potential to become a dominant bacterial group in the environment due to its low growth rate and its ability to utilize a variety of substrates [29]. However, the discovery of this strain is unpredictable, the number is small, and the diversity is low. Thus, it is not always possible to screen the target strains directly in polluted environments. Therefore, it is particularly important to further determine whether the isolated strains have degrading functions. Secondly, while the degradation function was revealed under laboratory conditions, it is worth further exploring whether the degradation function can be stabilized in the actual polluted environment.

Phenanthrene is a PAH with a tricyclic structure and is the smallest PAH molecule with both a Bay structure and a K region [30]. The bay region refers to a sterically hindered area formed between carbon atoms at positions 4 and 5, while the K-region represents the reactive double bond structure located between positions 9 and 10 in the phenanthrene molecule. These two distinct structural regions are widely recognized as the primary sites responsible for the carcinogenic activity of PAHs [31,32,33,34]. Due to its unique structural characteristics, moderate acute toxicity and genotoxicity, and widespread environmental distribution, phenanthrene has been extensively adopted as a model compound for PAH research. It serves as an ideal substrate for investigating various aspects of PAHs, including their toxicity profiles [35], transport and transformation mechanisms [36], and degradation pathways [37]. Particularly in biodegradation studies, phenanthrene is frequently utilized as a prototype compound for exploring the catabolic and metabolic processes of polycyclic aromatic hydrocarbons [20]. These attributes make it an ideal model for elucidating catabolic pathways applicable to broader PAH compounds. Therefore, this study was conducted with phenanthrene as the target pollutant.

In this study, we focused on the *Mycobacterium* sp., TJFP1, a strain previously isolated from coking wastewater during preliminary laboratory investigations. This strain demonstrated exceptional degradation efficiency towards phenanthrene, a model polycyclic aromatic hydrocarbon (PAH) compound. Through comprehensive performance evaluation combined with genomic analysis, we systematically investigated its degradation mechanisms. Furthermore, soil remediation simulations were conducted to assess the strain’s practical applicability, providing valuable theoretical insights for potential field implementation.

## 2. Materials and Methods

### 2.1. Chemicals and Culture Media

Phenanthrene (≥97%, purity, PHE), pyrene (≥98%, purity, PYR), fluorene (≥98% purity, FLN), anthracene (≥99%, purity, ANT), acenaphthene (≥98%, purity, ACE), fluoranthene (≥98%, purity, FLU) were purchased from Greagent (Shanghai Titan) Ltd., Shanghai, China); benzo(α)anthracene (≥98%, purity, BaA) was purchased from Aladdin (Shanghai Aladdin Biochemical Technology Co., Ltd., Shanghai, China); and benzo(α)pyrene (≥99%, purity, BaP) was purchased from Anpel (Shanghai Anpel Experimental Technology Co., Ltd., Shanghai, China).

Mineral salt medium (MSM) [38]: K_2_HPO_4_•3H_2_O 6.8 g/L, KH_2_PO_4_ 3.7 g/L, NH_4_Cl 2.0 g/L, (NH_4_)_2_SO_4_ 1.0 g/L, MgSO_4_ 0.1 g/L. An additional 1 mL of trace metal ion buffer per liter is required. In subsequent experiments, studies were conducted by adding appropriate amounts of PAHs to the MSM medium, thereby constructing a culture system with PAHs as the sole carbon source.

Trace metal ion buffer: FeCl_2_•4H_2_O 300 mg/L, Na_2_MoO_4_•2H_2_O 40 mg/L, CoCl_2_•6H_2_O 38 mg/L, MnCl_2_•4H_2_O 20 mg/L, ZnCl_2_ 14.0 mg/L, H_3_BO_3_ 12.4 mg/L, CuCl_2_•2H_2_O 3.4 mg/L.

Microbial enrichment culture medium was used with tryptic casein soybean peptone liquid (TSB) medium: casein tryptic digest 17 g/L, soybean meal papain digest 3 g/L; potassium dihydrogen phosphate 2.5 g/L; sodium chloride 5 g/L; glucose 2.5 g/L.

Phenanthrene liquor (1 g/L): 50.0 mg of phenanthrene solid was weighed and dissolved in 50 mL of ethyl acetate, filtered through a 0.22 μm organic phase filter membrane, and kept in a brown reagent bottle for spare use.

### 2.2. Strain Resource

TJFP1: obtained from the preliminary screening and isolation of the group in leather wastewater, initially identified as *Mycobacterium* sp. The strain was originally named F104.

The strain was conserved in the China Center for Type Culture Collection (CCTCC) under the conservation number CCTCC M 20242378.

### 2.3. Validation of the Degradation Function of Phenanthrene and Other PAHs by Stain TJFP1

A single colony of *Mycobacterium* sp., TJFP1, was picked into 50 mL of TSB liquid medium, and the shake flask was placed in a shaker at 30 °C and 200 rpm and incubated with shaking for 2 d and then taken out. After centrifugation at 4 °C and 8000 r/min for 10 min, the supernatant was removed, and the pure bacterial body was isolated, and the cell biomass was obtained, washed three times with MSM medium, and resuspended. Finally, the cell suspension was inoculated into culture medium for experiments.

Phenanthrene solution (1 g/L) was added to a pre-sterilized empty test tube sealed with a sealing film and placed in a fume hood. After waiting for the solvent to evaporate, 9 mL of sterilized MSM medium and 1 mL of the previously prepared suspension were added to the test tube. Thus, we constructed a phenanthrene degradation system with a phenanthrene concentration of 50 mg/L and a 10% strain inoculum. The test tubes were placed in a shaker at 30 °C and 200 rpm for 10 days of incubation with shaking. Samples were taken after 10 days to test the concentration of phenanthrene remaining in the degradation system and determine the degradation efficiency.

In the same process, strain TJFP1 was also tested for other PAHs (acenaphthylene (ACE), fluorene (FLN), anthracene (ANT), pyrene (PYR), fluoranthene (FLU), benzo[α]anthracene (BaA) and benzo[a]pyrene (BaP)) degradation efficiency. The initial concentration of all contaminants was set at 50 mg/L, and the degradation system was constructed as above.

Three parallels were set up for each group, while treatments without strains and with equal amounts of MSM medium added were used as blank control groups.

### 2.4. Detection and Quantification of Phenanthrene and Other PAHs

Extraction Method: Because of the water-insoluble nature of phenanthrene, the whole bottle extraction method was used. At the end of the strain culture, the test tube was removed, and an equal volume of ethyl acetate was added to the sample, vortexed, mixed well, and then sonicated in a water bath for 60 min. After waiting for 30 min, the upper organic phase and the lower aqueous phase were separated. The upper organic phase was filtered through a 0.22 μm organic phase filter membrane into a 2 mL brown injection vial and analyzed for phenanthrene.

**Figure A1:**
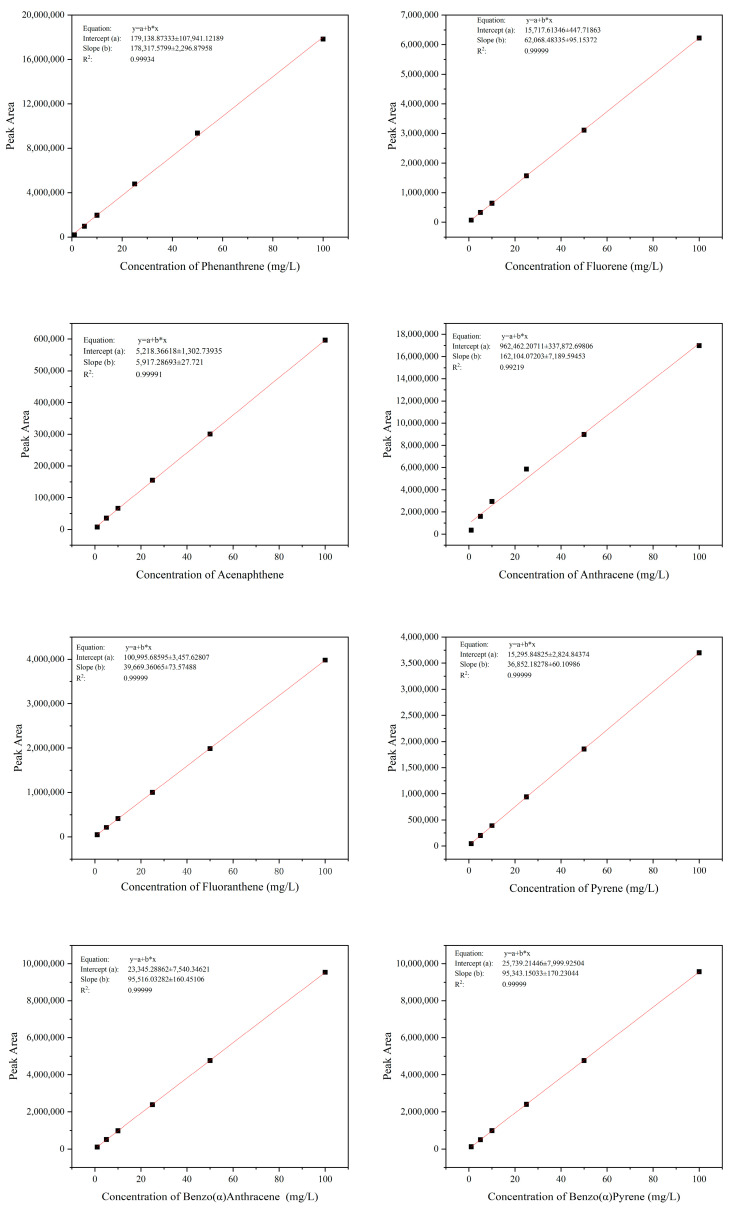
Standard curves for several PAHs.

Detection Methods: Chromatographic analysis was performed using a Waters (Milford, MA, USA) Premier UPLC system equipped with a TUV detector [35,39,40,41]. Separation was achieved on a Waters Symmetry C18 column (5 μm, 4.6 × 100 mm) maintained at 35 °C. The mobile phase consisted of acetonitrile and ultrapure water (85:15, *v*/*v*) delivered at a flow rate of 0.4 mL/min. Detection was carried out at 254 nm, with an injection volume of 5 μL. The retention times were determined by analyzing the peak elution times of various PAHs (PHE: 6.005 min; FLN: 5.992 min; ACE: 6.318 min; ANT: 6.985 min; FLU: 7.827 min; PYR: 8.767 min; BaA: 10.229 min; BaP: 5.723 min). The quantities in culture were calculated with the standard curve of each chemical. The R^2^ values for all standard curves were >0.99. We have provided the complete set of calibration curves for all target polycyclic aromatic hydrocarbons (PAHs) in Figure A1.

All reported concentrations reflect instrument-measured values rather than nominal spiking concentrations. Also, all initial concentrations of contaminants involved in the degradation efficiency calculations were replaced with the detected concentrations in the blank control group.

### 2.5. Effects of Environmental Conditions on the Degradation Performance of Stain TJFP1

A univariate experimental design was implemented to systematically evaluate the individual effects of incubation temperature, pH, and initial phenanthrene concentration on phenanthrene biodegradation efficiency. The initial concentration of phenanthrene was 50 mg/L in the temperature and pH experiments. For temperature optimization studies, cultures were incubated in a shaker at 20, 25, 30, and 37 °C for 10 days. To investigate pH effects, the medium pH was adjusted to values ranging from 4 to 10 (increments of 1 pH unit) using HCl and NaOH solutions, followed by incubation at 30 °C with 200 rpm shaking for 5 days. In the initial concentration experiments, phenanthrene was added to the medium at concentrations of 25, 50, 100, 200, and 500 mg/L. These cultures, prepared at pH 9, were incubated under identical conditions (30 °C, 200 rpm) for 5 days. The non-inoculated strains were used as control groups, and three parallels were set up in each group. At the end of the incubation cycle, biomass and residual phenanthrene were determined.

In addition, the relationship between the concentration of phenanthrene and the number of bacteria (OD_600_) and time was explored under optimal pH and temperature conditions at a concentration of 100 mg/L of phenanthrene in three parallel groups each.

### 2.6. Detection of Phenanthrene Biodegradation Intermediates

To determine the intermediate metabolites of phenanthrene biodegradation by strain TJFP1, the strain was inoculated into MSM medium with phenanthrene as the sole carbon source according to the above steps. Samples were taken at 0, 2, 5, and 10 d of incubation. After sample collection, the products were detected by gas chromatography-mass spectrometry (GC-MS) [35,39].

### 2.7. Whole Genome Sequencing and Annotation Analysis

Bacterial cultures were streaked onto sterilized Tryptic Soy Broth (TSB) agar plates for isolation. Well-isolated single colonies demonstrating robust growth were carefully selected and shipped on dry ice to Shanghai Genomics, Inc. (Shanghai, China) for whole genome sequencing analysis.

Genomic DNA was extracted and purified from the samples, followed by library preparation and sequencing. Whole genome sequencing was performed using a hybrid approach combining Whole Genome Shotgun (WGS) methodology with dual-platform sequencing (Illumina NovaSeq for second-generation sequencing and PacBio Sequel for third-generation sequencing, San Diego, CA, USA). Genomic libraries with varying insert sizes were constructed to ensure comprehensive genome coverage.

Protein-coding genes were predicted using GeneMarkS-2 software for genome annotation. tRNA genes were identified through tRNAscan-SE, while rRNA genes were detected using Barrnap. Additional non-coding RNAs were annotated through comparative analysis against the Rfam database. Functional annotation of protein-coding genes was conducted using multiple databases, including NR (Non-redundant Protein Sequence Database), KEGG (Kyoto Encyclopedia of Genes and Genomes), eggNOG (Evolutionary Genealogy of Genes: Non-supervised Orthologous Groups), Swiss-Prot, and GO (Gene Ontology).

### 2.8. Simulation of Soil Remediation Experiments

The soil remediation potential of strain TJFP1 was evaluated through a 20-day biodegradation experiment conducted under controlled laboratory conditions. The experimental design utilized 200 mL beakers, each containing 200 g of phenanthrene-contaminated soil. A 10% (*v*/*w*) bacterial suspension was aseptically introduced and thoroughly homogenized with the contaminated soil to ensure uniform distribution of the microbial inoculum. Two control groups were established for comparative analysis: (1) contaminated soil treated with an equivalent volume of sterile water, and (2) uncontaminated soil treated with sterile water. All experimental treatments were performed in triplicate to ensure methodological reproducibility and statistical reliability.

All experiments were conducted in a light-free artificial climate chamber maintained at 30 °C, with soil moisture content regulated between 15% and 20% throughout the incubation period. During the 20 days, soil samples (5 g) were collected at 5-day intervals for quantitative analysis of residual phenanthrene (PHE) concentrations.

The control group of virgin soil was named “NC” (No treatment Control Group), the control group of contaminated soil was named “PT” (Pollution Treatment Group), the control group of contaminated soil with sterile water was named “NG” (Natural degradation Group), and the experimental group of contaminated soil inoculated with strain TJFP1 was named “BG” (Biodegradation Group).

Multiple soil samples were collected at different stages to evaluate the changes in soil physicochemical properties and microbial community structure during the biodegradation process comprehensively. Initial samples included pristine soil (NC) and phenanthrene-contaminated soil (PT), collected prior to the experiment to establish baseline characteristics and assess the impact of phenanthrene contamination. Subsequently, soil samples from both the natural degradation group (NG) and the experimental group (BG) were collected at 5-day and 20-day intervals following the initiation of the simulated remediation experiment. This sampling strategy enabled comparative analysis of natural remediation processes versus enhanced bioremediation effects on soil properties.

The samples were collected at each sampling time point and immediately stored at −80 °C in an ultra-low temperature freezer to preserve sample integrity until analysis. Then, they were sent to the Shanghai Majorbio Bio-pharm Technology Co., Ltd. for microbial diversity analysis using high-throughput sequencing technology.

## 3. Results

### 3.1. Determination of the Degradation Function of Strain TJFP1 on Phenanthrene and Other PAHs

The degradation efficiencies of common PAHs by the *Mycobacterium* sp., TJFP1, after 10 days at 30 °C with 200 rpm agitation are presented in Table 1. According to the results, it can be found that strain TJFP1 has a certain degradation effect on three-ring PAHs (PHE, ANT, ACE, FLN), and part of four-ring PAHs (FLU, PYR), and some of the samples are able to realize complete degradation. The experimental data suggest that the strain’s intrinsic enzymatic degradation system demonstrates preferential activity towards low molecular weight PAHs with relatively simple structural configurations [42]. PAHs are typically difficult to degrade, and their bioavailability generally decreases as the number of benzene rings increases [43]. Although the structures of these PAHs may be similar, strain TJFP1 showed great differences in their degradation effects. This may be due to the fact that bacteria have a more stringent transcriptional regulatory system, and compounds that differ slightly from the substrate do not induce the expression of the relevant degradative enzyme system [44].

**Figure 1:**
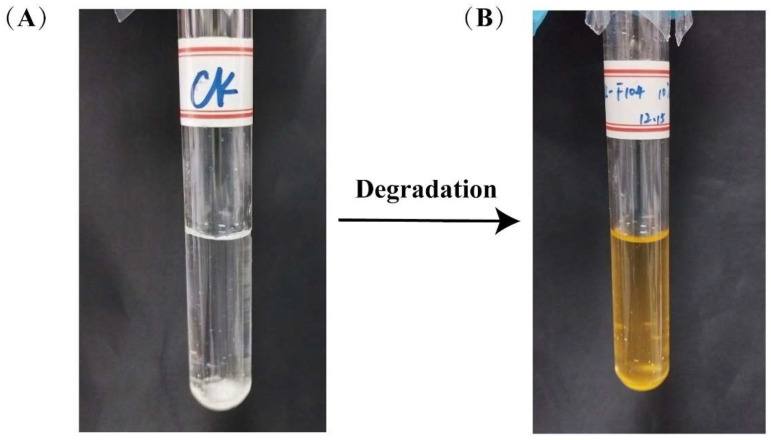
Phenanthrene degradation system medium color change: (**A**) pre-degradation; (**B**) after degradation.

Meanwhile, noticeable color changes were observed in all media capable of supporting degradation. Particularly in the phenanthrene degradation system, the media transitioned from an initial colorless and transparent state to a gradually deepening hue as incubation progressed, ultimately stabilizing to a reddish-brown color (Figure 1). This phenomenon may be attributed to structural alterations in the compounds during the degradation process or the accumulation of intermediate metabolites [27,35].

In summary, the results demonstrate that strain TJFP1 exhibits a broad substrate degradation spectrum, with the capability to degrade most typical polycyclic aromatic hydrocarbons (PAHs). Furthermore, strain TJFP1 exhibited significantly enhanced degradation efficiency towards phenanthrene compared to certain previously reported phenanthrene-degrading strains and microbial consortia [45,46,47].

### 3.2. Effect of Temperature, pH, and Initial Concentration on Degradation

**Figure 2:**
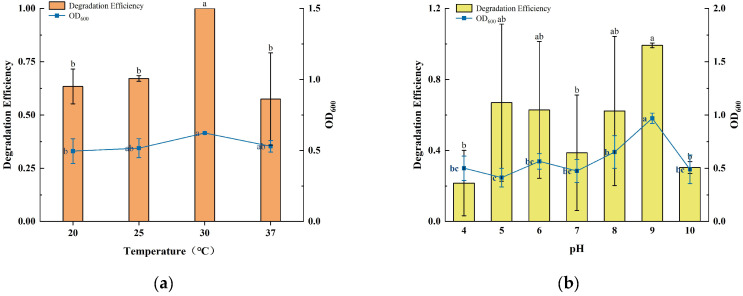

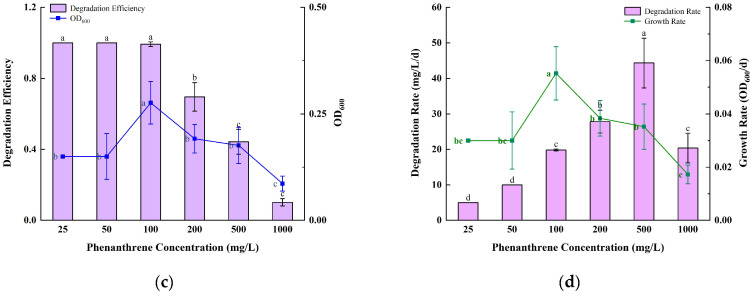
Growth and degradation of TJFP1 under different environmental conditions: (**a**) Different Temperature; (**b**) Different pH; (**c**) Different Initial phenanthrene concentration; (**d**) Growth and degradation rates at different initial concentrations. The significance of the difference between the different groups in each figure is indicated by letter labeling, with the same letter indicating no statistical difference between the groups and different letters indicating a significant difference (*p* > 0.05).

Environmental factors such as temperature, pH, and initial concentration had a significant effect on the efficiency of microbial degradation of PAHs. The growth of strain TJFP1 and the degradation efficiency of phenanthrene under different conditions are shown in Figure 2.

At different incubation temperatures, strain TJFP1 showed significant differences in the degradation of phenanthrene as well as in the growth status. The OD_600_ of strain TJFP1 at 30 °C was significantly higher than that at other temperatures. Thus, it can be hypothesized that the reproduction rate of TJFP1 was accelerated and the metabolic activity was significantly enhanced at 30 °C relative to other temperature conditions. This result is consistent with the degradation efficiency, further validating the effectiveness of the degradation process. Throughout the 10-day degradation period, strain TJFP1 completely degraded phenanthrene, achieving a 100% degradation efficiency in all three replicate groups at 30 °C. However, at other temperatures, the degradation efficiency is around 60%. Clearly, strain TJFP1 exhibited optimal phenanthrene degradation efficiency at 30 °C.

At different culture pH levels, the degradation period was reduced to 5 days to highlight the degradation differences of phenanthrene. It was observed that both the degradation efficiency and the growth state of strain TJFP1 varied significantly with changes in pH. The highest degradation efficiency of 100% and the optimal growth state were achieved at pH 9. Except for pH 4, the average degradation efficiency at all other pH levels exceeded 30%, with no significant differences observed in growth status.

To further investigate the degradation capability of strain TJFP1, phenanthrene at varying concentrations (25, 50, 100, 200, and 500 mg/L) was added to the inorganic salt medium as the sole carbon source. Subsequently, the growth of the strain and the degradation efficiency were assessed. When considering only the degradation efficiency, strain TJFP1 was capable of degrading phenanthrene (PHE) within 5 days when the initial PHE concentration was less than 100 mg/L. The degradation efficiency decreases with increasing initial substrate concentration. While considering the average degradation efficiency per unit time (i.e., degradation rate), the highest average degradation rate (44.32 mg/L·d^−1^) was observed when the initial PHE concentration was 500 mg/L. It can also be found that the lower initial substrate concentration corresponds to a lower average degradation rate, which increases to some extent as the concentration increases. However, when the PHE concentration increases to a certain level (>500 mg/L), the degradation rate decreases. OD_600_, an indicator reflecting the growth status of the strain, and the growth rate per unit time (OD_600_/d) exhibited consistent trends. The difference was that strain TJFP1 had the fastest growth rate when the initial concentration of PHE was 100 mg/L, which produced a significant difference from the other concentrations. It is also evident that during the biodegradation of pollutants, microorganisms exhibit varying degradation capacities for different concentrations of the same substrate, which is not entirely synchronized with their growth state [48].

Substrate concentration plays a critical role in bacterial growth and enzymatic activity. While excessively low concentrations fail to support bacterial survival or induce degradative enzymes, excessively high concentrations, despite triggering the expression of degradative genes, may exert toxic effects on microbial cells, ultimately inhibiting bacterial growth [49,50].

Despite variations in degradation efficiency and growth patterns, strain TJFP1 demonstrated significant phenanthrene degradation capabilities across a broad range of temperature and pH conditions. This remarkable environmental adaptability distinguishes TJFP1 from many other phenanthrene-degrading bacterial strains.

### 3.3. Growth and Degradation Curves of Strain TJFP1 for the Degradation of 100 mg/L Phenanthrene at Optimal Temperature and pH

**Figure 3:**
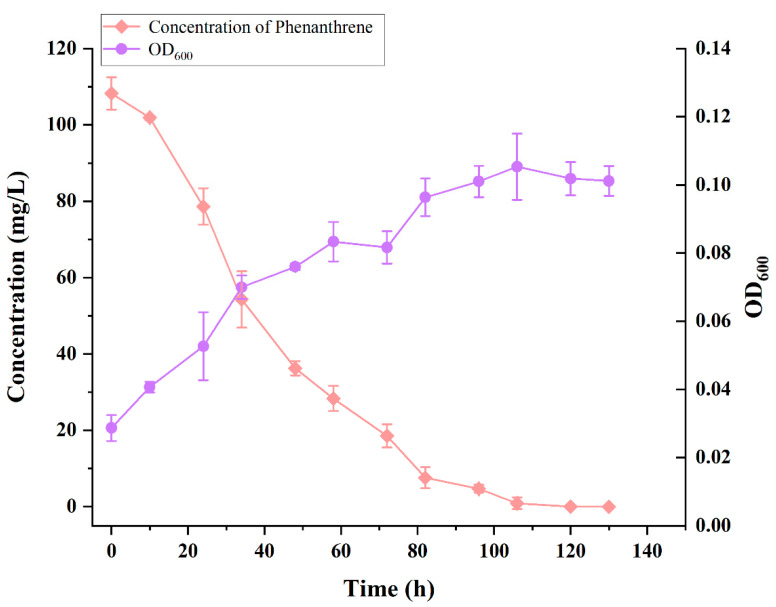
Growth and degradation curves of strain TJFP1.

The OD_600_ of strain TJFP1 and the concentration of remaining phenanthrene in the culture system with time at an incubation temperature of 30 °C, pH 9, and an initial concentration of 100 mg/L of phenanthrene are shown in Figure 3.

Strain TJFP1 entered the logarithmic growth phase at 24 h (1 day), reaching its maximum growth rate. The growth rate began to decrease at 82 h (approximately 3.5 days), and the strain reached the stationary phase at 106 h (about 4.5 days), with the final biomass reaching an OD_600_ of 0.115. During the bacterial growth process, the PHE concentration in the MSM medium decreased continuously. The degradation process progressed synchronously with bacterial growth, and the initial 100 mg/L PHE (the actual detected initial concentration was 108.24 ± 2.12 mg/L) was almost completely degraded after 106 h (4.5 days) of cultivation. Compared to most of the other reported strains (see Table 2), strain TJFP1 degraded phenanthrene at a higher rate and with a shorter degradation cycle.

### 3.4. Whole Genome Sequencing Analysis

The whole genome sequencing of strain TJFP1 was performed using both Illumina Novaseq and PacBio Sequel high-throughput sequencing platforms. Initial sequencing yielded 6,522,216 raw reads, comprising 978,332,400 bases. After quality control, 6,092,100 high-quality reads containing 906,679,979 high-quality bases were obtained. Data analysis revealed a GC content of 67.22% in the clean reads. Quality assessment demonstrated that the proportions of bases with Q20 (accuracy ≥ 99%) and Q30 (accuracy ≥ 99.9%) reached 98.14% and 94.73%, respectively, confirming the high accuracy and reliability of the sequencing results.

**Figure 4:**
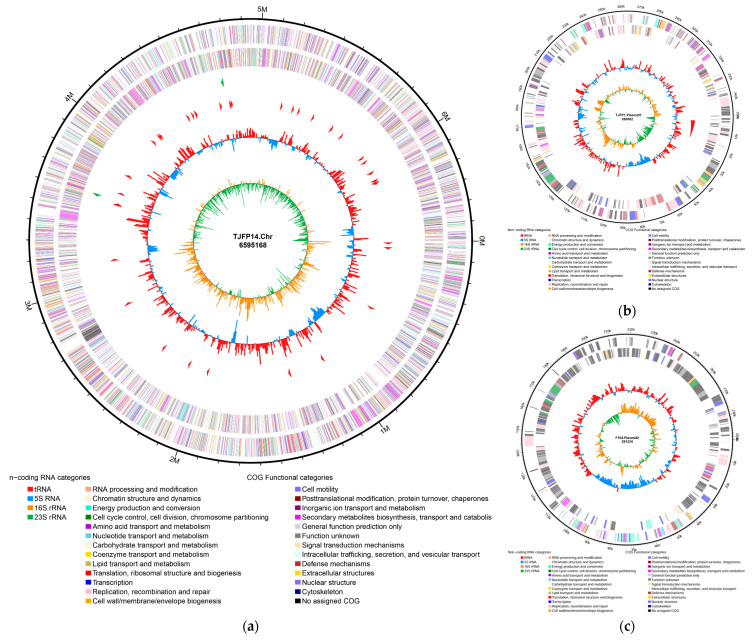
Genome circle map of strain TJFP1: (**a**) Chromosome; (**b**) Plasmid1; (**c**) Plasmid2. The outermost circle represents the genome size scale. The second and third circles display CDS (Coding Sequences) on the forward and reverse strands, respectively, with different colors indicating distinct COG (Clusters of Orthologous Groups) functional classifications. The fourth circle illustrates the distribution of rRNA and tRNA. The fifth circle depicts GC content, where outward red peaks indicate regions with GC content higher than the genome average (the height of peaks corresponds to the degree of deviation from the average), and inward blue peaks represent regions with GC content lower than the genome average (similarly, peak height reflects the extent of deviation). The innermost circle shows the GC skew: (G − C)/(G + C).

Genome assembly results revealed that strain TJFP1 possesses a total genome size of 7,237,284 bp with an N50 value of 6,595,168 bp and an overall GC content of 67.46%. The assembly yielded one circular genome and two linear genomes (Figure 4): the circular genome represents the chromosome of strain TJFP1, measuring 6,595,168 bp with a GC content of 67.70%; the two linear plasmids were identified as plasmid 1 (350,882 bp, 64.65% GC content) and plasmid 2 (291,234 bp, 65.36% GC content).

The predicted protein sequences were aligned against multiple databases, including NR, Swiss-Prot, eggNOG, KEGG, GO, CARD, and CAZy, to obtain functional annotations for the predicted genes. The functional annotation results of protein-coding genes across these databases are summarized in Table 3.

**Figure 5:**
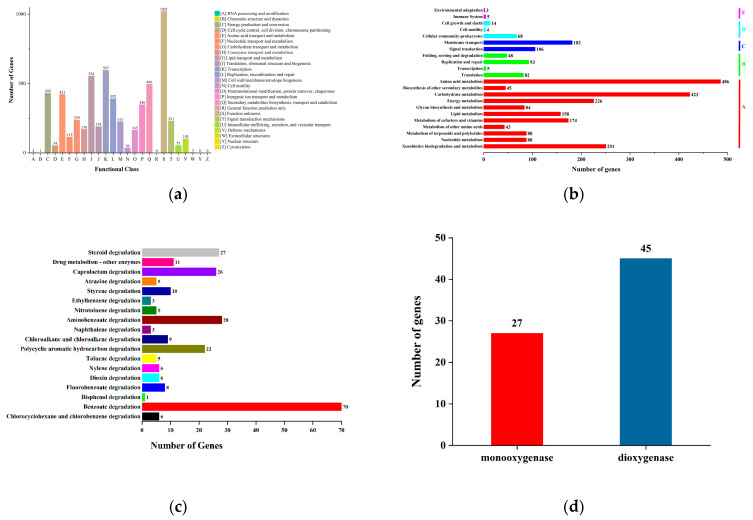
Annotated classification of strain TJFP1 genes: (**a**) COG functional categories; (**b**) KEGG annotation classification; (**c**) Annotation of genes involved in exogenous degradation and metabolic pathways in strain TJFP1; (**d**) Number of oxygenase genes contained in strain TJFP1.

The COG annotation results for the genes were obtained through DIAMOND alignment with the eggNOG database. Based on the COG annotations, the proteins were functionally categorized, as illustrated in Figure 5a. The six most abundant functional groups were: Group S (1021 genes, function unknown); Group K (597 genes, Transcription); Group I (554 genes, Lipid transport and metabolism); Group Q (496 genes, Secondary metabolites biosynthesis, transport, and catabolism); Group C (432 genes, Energy production and conversion); Group E (421 genes, Amino acid transport and metabolism).

Meanwhile, through functional annotation and prediction of genes in strain TJFP1, combined with comparisons to known genes in the NCBI database, the genes were categorized according to the KEGG database, as shown in Figure 5b. A total of 2832 annotated genes were classified into five functional categories: Metabolism (A); Genetic Information Processing (B); Environmental Information Processing (C); Cellular Processes (D); Organismal Systems (E). Functional gene annotation revealed that the largest proportion of genes belonged to the “metabolism” category (72.95%), followed by “environmental information processing” (10.17%), “genetic information processing” (8.05%), “cellular processes” (3.04%), and “organismal systems” (0.28%). Within the “metabolism” category, the subcategories with the highest gene counts were as follows: Amino acid metabolism (486 genes); Carbohydrate metabolism (423 genes).

Notably, strain TJFP1 possesses a significant number of genes (251) involved in “xenobiotic biodegradation and metabolism.” These genes account for 12.15% of the genes in the “metabolism” category and 8.86% of the total annotated genes. This finding highlights the strain’s strong potential for bioremediation applications. The genome of strain TJFP1 was further analyzed to identify genes associated with “xenobiotic biodegradation and metabolism”. The distribution of genes across various degradation pathways is illustrated in Figure 5c. Among the 251 genes identified, the majority were involved in the biodegradation pathways of polycyclic aromatic hydrocarbons (PAHs) and other organic compounds containing benzene ring structures, underscoring the strain’s specialized metabolic capabilities. For instance, strain TJFP1 harbors 70 genes associated with benzoate degradation, 28 genes involved in aminobenzoate degradation, and 22 genes linked to polycyclic aromatic hydrocarbon (PAH) degradation. Additionally, genes related to styrene degradation (10) and naphthalene degradation (3) were identified. Collectively, these genes account for 66.53% of the total genes involved in xenobiotic biodegradation and metabolism, highlighting the strain’s significant capacity for degrading aromatic compounds.

These degradation pathways are closely associated with the biodegradation of aromatic compounds, including benzoate, toluene, xylene, and ethylbenzene. These compounds are aromatic hydrocarbons containing benzene ring structures and are often downstream metabolic analogs of PAHs [44,70,71]. This specificity in degradation pathways demonstrates that strain TJFP1 exhibits a high degree of specialization for the degradation of PAHs and related aromatic compounds.

Furthermore, studies have demonstrated that the microbial degradation of polycyclic aromatic hydrocarbons (PAHs) is initiated by the oxidative cleavage of the benzene ring structure, a critical step catalyzed by oxygenase enzymes, which play an indispensable role in PAH degradation pathways [72,73]. Given that bacterial degradation of polycyclic aromatic hydrocarbons (PAHs) primarily involves dioxygenase-mediated reactions, with some contributions from monooxygenase-mediated processes [74,75,76], the gene annotation results were systematically screened using the keywords “monooxygenase” and “dioxygenase” to identify relevant enzymatic components. A total of 27 monooxygenase genes and 45 dioxygenase genes were identified, as illustrated in Figure 5d. This genetic repertoire underscores the significant potential of strain TJFP1 for PAH degradation, highlighting its robust enzymatic machinery for breaking down these persistent environmental pollutants.

### 3.5. Pathway of Phenanthrene Degradation by Strain TJFP1

**Figure 6:**
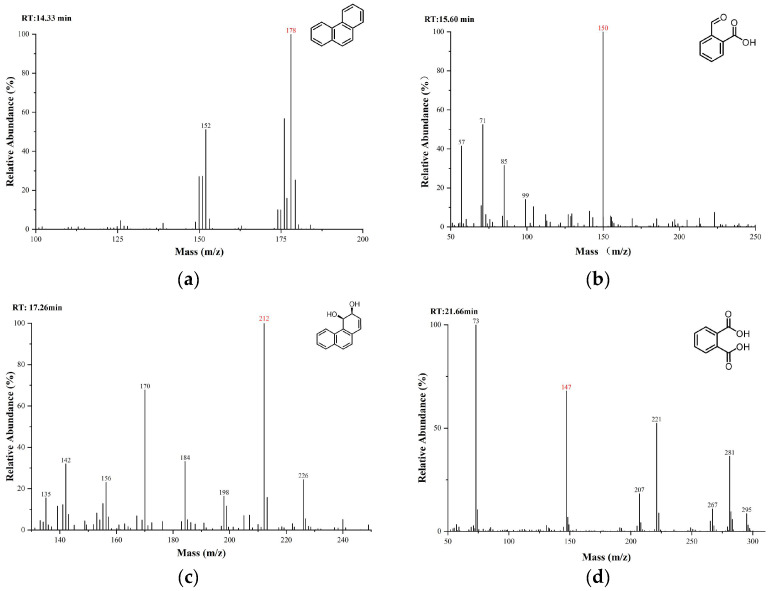
Identification of intermediate metabolites in the degradation of phenanthrene and phenanthrene: (**a**) phenanthrene; (**b**) 2-Carboxybenzaldehyde; (**c**) cis-3,4-Dihydroxy-3,4-dihydrophenanthrene; (**d**) phthalic acid (BSTFA-derivatized).

The substances in the culture medium were initially analyzed using gas chromatography-mass spectrometry (GC-MS). The total ion chromatograms (TICs) of the intermediate metabolites were obtained by comparing samples collected at different time points and BSTFA-derivatized samples with the 0-day control, as shown in Figure 6. By comparing the mass spectra with the NIST MS database, MS spectra of standard compounds, and intermediate product spectra reported in the literature, combined with the molecular ion peak values, the detected compounds were tentatively identified as 2-carboxybenzaldehyde Figure 6b, cis-3,4-dihydroxy-3,4-dihydrophenanthrene Figure 6c, and derivatized phthalic acid Figure 6c. These are all common intermediates in the biodegradation of phenanthrene.

**Figure 7:**
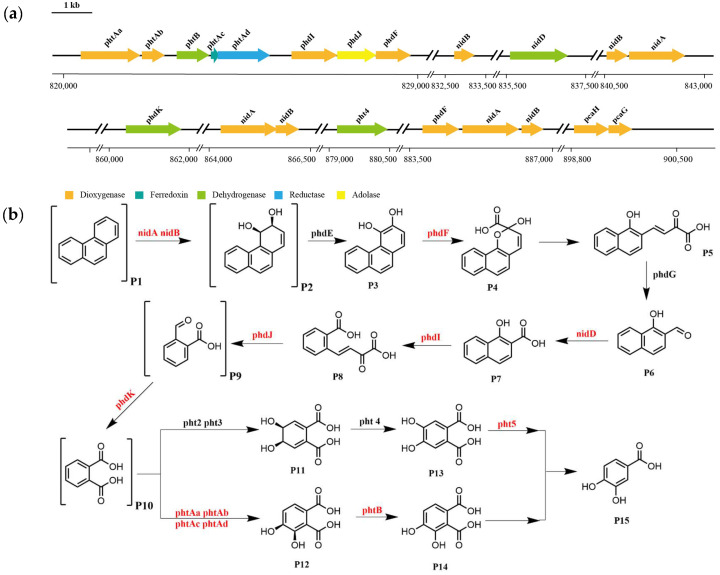
Key genes and degradation pathways for phenanthrene degradation by strain TJFP1: (**a**) Genes related to phenanthrene degradation and location; (**b**) Presumed degradation pathways. Substances labeled in parentheses are detected metabolites, and genes labeled in red are genes detected and annotated in strain TJFP1. P1: Phenanthrene; P2: Phenanthrene cis-3,4-dihydrodiol; P3: 3,4-Dihydroxyphenanthrene; P4: 2-Hydroxy-2H-benzo[h]chromene-2-carboxylate; P5: Cis-4-(1′-Hydroxynaphthalen-2′-yl)-2-oxobut-3-enoate; P6: 1-Hydroxy-2-naphthaldehyde; P7: 1-Hydroxy-2-naphthoic acid; P8: Cis-2′-Carboxybenzalpyruvate; P9: 2-Carboxybenzaldehyde; P10: Phthalic acid; P11: Phthalate-4,5-cis-dihydrodiol; P12: Phthalate-3,4-cis-dihydrodiol; P13: 4,5-Dihydroxyphthalic acid; P14:3,4-Dihydroxyphthalic acid; P15: Protocatechuic acid.

Based on the functional annotation and prediction of strain TJFP1’s genes, combined with the degradation pathways identified through KEGG analysis, it was revealed that TJFP1 harbors a diverse array of genes encoding enzymes for PAH degradation. Although certain genes involved in phenanthrene metabolism appear to be missing from the degradation pathway, potentially due to genetic differences between strain TJFP1 and typical PAH-degrading microorganisms [77], the overall pathway contains a relatively complete set of functional genes for phenanthrene degradation. Genomic analysis revealed the clustered organization of phenanthrene metabolic enzyme genes on the chromosome of strain TJFP1 (Figure 7a and Table A1).

Based on whole-genome sequencing of strain TJFP1 and KEGG pathway analysis, combined with the detection of intermediate metabolites, we propose a putative metabolic pathway for phenanthrene degradation, as illustrated in Figure 7b. The results suggest that strain TJFP1 likely degrades phenanthrene via the phthalic acid pathway [26,27,78]. Building upon previous studies, we propose that, similar to aerobic degradation of other PAHs, phenanthrene catabolism in strain TJFP1 is initiated by dioxygenase-catalyzed oxidation. Strain TJFP1 initiates phenanthrene degradation through dioxygenase-mediated (nidA, nidB) primary oxidation at the C-3 and C-4 positions of the aromatic ring [79,80]. Subsequently, the intermediate is dehydrogenated by a ring-hydroxylating dehydrogenase to form phenanthrene cis-3,4-dihydrodiol [81]. This product is further oxidized by PhdF to yield cis-3,4-dihydroxyphenanthrene [82]. These intermediate metabolites subsequently undergo sequential enzymatic oxidation, ultimately yielding 1-hydroxy-2-naphthaldehyde as a key degradation product [83]. The 1-hydroxy-2-naphthaldehyde is subsequently oxidized to 1-hydroxy-2-naphthoic acid by the NAD+-dependent aldehyde dehydrogenase nidD [78,79]. Notably, 1-hydroxy-2-naphthoic acid serves as a critical metabolic marker, indicating the completion of the first ring cleavage reaction in the phenanthrene degradation pathway. Following the initial ring cleavage, cis-2′-carboxybenzalpyruvate is generated through phdI-mediated dioxygenation. This intermediate subsequently undergoes fission and condensation to form 2-carboxybenzaldehyde [84], which is further metabolized via the phthalate and protocatechuate pathways before ultimately entering the TCA cycle [80,85]. Finally, this metabolic cascade achieves complete mineralization of phenanthrene.

### 3.6. Assessment of Remediation Efficacy and Microbial Community Response to Phenanthrene-Contaminated Soil Driven by Strain TJFP1

#### 3.6.1. Strain TJFP1 Is Able to Produce Remediation Effects on Phenanthrene-Contaminated Soil

**Figure 8:**
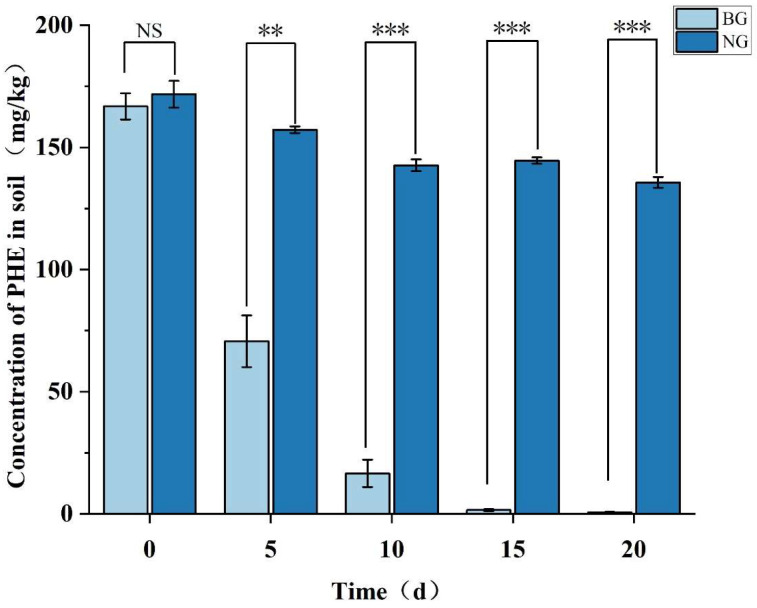
Concentration of residual PHE during soil bioremediation and natural remediation. Significance levels: *** *p* < 0.001, ** *p* < 0.01; NS, not significant (*p* ≥ 0.05).

Strain TJFP1 was added to the phenanthrene-contaminated soil for a twenty-day soil remediation trial, with regular sampling every five days to detect phenanthrene concentrations in the soil using UPLC, thereby assessing the remediation capacity of strain TJFP1. Comparison of the degradation of phenanthrene in the soil of the two treatment groups, NG and BG, is shown in Figure 8.

The initial phenanthrene (PHE) concentration in the soil was 171.81 mg/kg. Monitoring at different time points revealed that the natural attenuation control group (NG group) showed a 21.01% reduction in PHE concentration after 20 days of remediation. This reduction may be attributed to multiple physical and biological processes, including volatilization, soil adsorption, and biodegradation by indigenous soil microorganisms [86]. In contrast, samples inoculated with *Mycobacterium* TJFP1 (BG group) demonstrated significantly enhanced PHE degradation, with concentrations declining to 42.36% of initial levels by Day 5. The degradation progressed rapidly, reaching 9.94% by Day 10 and achieving 99.66% removal efficiency by Day 20. This was a marked improvement over natural attenuation by indigenous soil microorganisms alone. Thus, it can be judged that the significant reduction in PHE concentration in the soil of the BG group is the result of bioremediation dominated by strain TJFP1.

#### 3.6.2. Changes in Microbial Community Structure and Diversity

**Figure 9:**
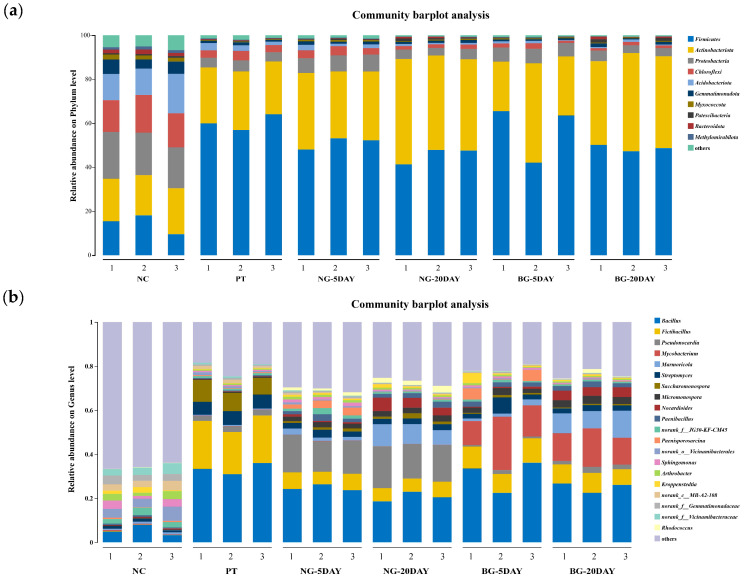
Modification of microbial communities under natural remediation and strain TJFP1-driven bioremediation of phenanthrene-contaminated soil: (**a**) Relative abundance at phylum level > 1%; (**b**) Top 20 genera with genus-level relative abundance greater than 1%.

Consistent with previous findings [15,86,87,88,89], the uncontaminated soil exhibited a balanced microbial community structure with optimal functional diversity. However, phenanthrene contamination significantly disrupted this equilibrium, resulting in the following. (1) Reduced α-diversity (Table 4), i.e., species diversity and richness decreased significantly: the Shannon index decreased by 40.6% (indicating significant decline in diversity); the Simpson index increased by 886% (showing dramatic rise in dominance and severe reduction in diversity); the ACE index declined by 19.7% (reflecting reduced species richness); the Chao1 index dropped by 19.2% (demonstrating decrease in total species number); the Pielou index fell by 38.1% (suggesting impaired community evenness). (2) Community structure shifts (Figure 9, Table A2): Actinobacteria increased from 19.63% to 25.36%, Firmicutes increased from 14.42% to 60.32%. (3) Dominance establishment: these phyla became the predominant taxa, collectively representing >85% of the total community.

In the absence of *Mycobacterium* TJFP1 inoculation, natural attenuation over 5 and 20 days revealed the following microbial shifts: Firmicutes remained the most abundant phylum but exhibited a gradual decline in relative abundance (Figure 9a and Table A2). Actinobacteria progressively increased, reaching near-equilibrium with Firmicutes after 20 days. The combined average relative abundance of these two phyla reached 89.69% at day 20 (Figure 9a and Table A2). At the genus level, the dominant soil genera shifted from *Bacliius* (30.91–33.36%) and *Fictibacillus* (19.20–21.71%) to *Bacliius* (18.58–22.94%) and *Pseudonocardia* (15.66–18.95%) by 20 days of natural degradation Figure 9b and Table A3. These observations suggest that: (1) Firmicutes and Actinobacteria emerge as the dominant populations in phenanthrene-contaminated soil, demonstrating significant contaminant tolerance. (2) Indigenous microorganisms of the genus Pseudonocardia are important in the process of natural soil remediation. (3) Correlated with the observed decline in soil phenanthrene concentrations (Figure 8), these microorganisms likely contribute to the natural degradation process.

Changes at the phylum level in soil bioremediation community composition with the addition of strain TJFP1 were similar to bioremediation, with the highest abundance of Firmicutes in the soil, followed by Actinobacteria, and the two percentages leveled out over time, totaling above 80% (Figure 9a and Table A2). At the genus level, in the “BG-5DAY” and “BG-20DAY” groups, to which strain TJFP1 was added for bioremediation, it was observed that *Mycobacterium* sp. increased from nearly was absent to the second most abundant genus (10.84–24.23% in the “BG-5DAY” group and 12.18–17.46% in the “BG-20DAY” group), and became the absolutely dominant genus (Figure 9b and Table A3). Corresponding to the above experimental results, *Mycobacterium* TJFP1 was successfully introduced into the remediation system and exerted a degrading effect. At the same time, the abundance of indigenous functional bacterial genera *Pseudonocardia* and *Bacillus*, which dominate the natural restoration, was significantly reduced, suggesting that the introduction of exogenous strains may have disturbed the indigenous community through competition for resources or ecological niche extrusion.

It is known from the above (Figure 8) that after 20 days of remediation with the addition of strain TJFP1, the phenanthrene in the soil was basically completely degraded, but the difference between the proportion of microorganisms in the soil and that in the uncontaminated (“NC” group) was still very obvious (Figure 9), so it can be seen that even if the pollutants are basically removed, the diversity of the soil microbial community is not able to be restored quickly. This is also consistent with Table 4. The effect of passive remediation is limited by the fact that natural remediation continues to decline in diversity over time, whereas natural remediation suppresses diversity in the short term, but in the long term, it can alleviate pollution pressures and lead to a gradual ecological recovery.

## 4. Discussion

This study centered on the functional analysis of phenanthrene-degrading bacteria and the potential for environmental remediation, using the *Mycobacterium* sp., TJFP1, as a research target. We revealed its potential for application in the treatment of polycyclic aromatic hydrocarbons (PAHs) pollution through multidimensional experiments.

In this study, it was first determined that strain TJFP1 showed different degrees of degradation ability for low molecular weight PAHs such as phenanthrene, fluorene, and anthracene, suggesting that it has a broad-spectrum degradation potential. This contrasts sharply with specialized degraders like *Pseudomonas stutzeri* ZP2, which fails to metabolize anthracene [90]. In actual contaminated environments, which are commonly a mixed source of multiple low and high-molecular-weight aromatic compounds, strain TJFP1, which has a broad substrate spectrum, is more suitable for actual environmental remediation. The strain’s catabolic flexibility may stem from its evolutionary adaptation to coking wastewater, an environment typically containing complex hydrocarbon mixtures.

Meanwhile, the change in environmental conditions showed that the strain had excellent resistance properties, being able to grow and degrade phenanthrene at different temperatures (20–37 °C) and pH (4–10). This characteristic of strain TJFP1 was significantly better than that of other degrading microorganisms. Numerous strains were highly sensitive to altered environmental conditions. For example, *Aquabacter sediminis* P-9T had the highest activity in degrading phenanthrene at 37 °C and was able to degrade phenanthrene completely, while the degradation efficiency was <20% at the same time when the temperature was lowered to 20 °C. At the same time, at pH 9, the strain showed little degradation of phenanthrene, but at pH 7.0, it was almost completely degraded in 5 days [91]. The *PseudArthrobacter* sp., L1SW, completely degraded 250 mg/L of phenanthrene within 36 h at pH 7.4, but phenanthrene degradation decreased to 20% at pH 5.0 [41]. *Pseudomonas* sp. Lphe-2 degradation rate as low as 10% at pH 8 [36]. These robust phenotypic characteristics prompted us to investigate the genetic basis underlying TJFP1′s exceptional performance, particularly in comparison to well-characterized PAH degraders.

Furthermore, the influence of substrate concentration on microbial growth and degradation efficiency was systematically evaluated. Although most of the degrading functional bacteria were acclimated to contaminants, they typically exhibit growth inhibition at elevated concentrations. This phenomenon may result from either: (1) alterations in transmembrane transport mechanisms leading to intracellular substrate accumulation at lower concentrations [90], or direct inhibitory effects of high substrate concentration [90,92]. For instance, the relatively slow degradation of *Mycolicibacterium* sp. Pyr9 at pollutant concentrations above 100 mg/L may be attributed to the inhibition of degradation due to the toxic effect of the high concentration of pollution on Pyr9 [92]. However, TJFP1 also showed high tolerance to phenanthrene, and could grow and degrade phenanthrene at an initial concentration of 1 g/L. The tolerance of the strain TJFP1 to environmental conditions, as well as substrate concentrations, means that it can survive in complex, contaminated environments without compromising its environmental function.

Additionally, genomic analysis of strain TJFP1 revealed multiple gene clusters associated with PAH degradation, predominantly located on its chromosome. The first PAHs degradation genes identified were from the nah gene cluster on the *Pseudomonas putida* G7 large plasmid NAH7 [84], which is responsible for coding the enzymes required for the upstream degradation pathway of naphthalene. In contrast, the phenanthrene degradation genes nid and phd genes mined in this study are conserved features of PAH-metabolizing bacteria of the *Mycobacteriaceae* family, which are functionally identical to the classical nah-like genes but do not possess homology. Further analysis determined that strain TJFP1 degraded phenanthrene following the traditional phthalic acid pathway [48]. The genomic characterization of TJFP1 provides crucial insights into PAH metabolic networks and establishes a foundation for mechanistic studies of bacterial PAH degradation.

The simulated soil remediation experiments of TJFP1 have further verified its on-site remediation efficacy and potential for engineering application. Various studies in the past have shown that direct introduction of degrading bacteria into the field does not always produce satisfactory remediation results [86]. Harsh environments [93], nutrient deprivation [94], and antagonism by native microorganisms [95] would all limit the effectiveness of bioremediation. Introduction of *Mycobacterium* TJFP1 as an exotic agent into soil ecosystems is a bioinvasive process that may disrupt native microbial communities by altering stochastic and deterministic assembly mechanisms [96]. In other reports, such perturbations have been shown to lead to loss of diversity, thereby affecting the efficacy of bioremediation [97]. In contrast, our results showed that the dramatic changes in the microbial community induced by TJFP1 inoculation, including the competitive exclusion of native degraders (Pseudonocardia and Bacillus) and the establishment of dominance of the introduced strains, did not jeopardize the degradation efficiency of phenanthrene. Despite significant community simplification, the removal of phenanthrene was essentially close to 100%. This suggests that functional redundancy through the powerful catabolic mechanism of TJFP1 (phd/nid genes) compensates for the reduction in taxonomic diversity, highlighting the environmental dependence between microbial diversity and ecosystem function in polluted environments. Collectively, our findings reveal two critical insights: (1) microbial community alterations persist post-remediation, indicating long-term ecological impacts of contamination; and (2) functional redundancy can outweigh diversity effects in pollutant degradation, providing new perspectives for bioaugmentation strategies.

Although the PAH degradation potential of *Mycobacterium branchiense* has been revealed in other previous studies, TJFP1 showed a more prominent degradation function. For example, the *Mycolicibacterium* sp., SCSIO 43805, required 12 days to effectively degrade pyrene and phenanthrene at a concentration of 50 mg/L. The results of this study are summarized in the following table [98]. Also, its remediation in soil was superior to that reported in other studies, where *Mycobacterium* spp. NJS-1 and NJS-P were introduced into contaminated soil, and only 30% of phenanthrene was degraded in one study [99]. These indicate that strain TJFP1 is a bioresource with strong potential for environmental remediation applications.

Subsequently, the functions of key genes and their synergistic mechanisms can be clarified through transcriptomics, proteomics, and gene knockdown experiments for in-depth analysis of the degradation mechanism. The potential for further degradation of high molecular weight PAHs can also be explored through macro-genomics or functional screening and gene integration with other degrading strains to construct a composite degradation pathway.

## 5. Conclusions

In conclusion, this study illustrated the degradation characteristics of TJFP1 on PAHs (mainly phenanthrene) with a broad substrate spectrum for typical PAHs, and revealed the genetic basis of PAH degradation by this strain through whole genome sequence analysis, predicted the metabolic pathway of phenanthrene, and at the same time evaluated the feasibility of its engineering application through soil remediation simulation experiments, which provided the field of PAH remediation with exploitable bioresources.

## Figures and Tables

**Table 1:** Degradation efficiencies of polycyclic aromatic hydrocarbons (PAHs) by the *Mycobacterium* sp., TJFP1, in mineral salts medium (MSM) at 30 °C with 200 rpm agitation for 10 days.

PAHs	Degradation Ability *	Degradation Efficiency **	Medium Color Change ***
PHE	++	89.82 ± 15.17%	+
ANT	+	20.78 ± 18.36%	+
ACE	++	81.3 ± 2.73%	+
FLN	++	100%	+
FLU	+	20.48 ± 4.36%	+
PYR	+	23.72 ± 15.03%	+
BaA	-	3.8 ± 2.87%	-
BaP	-	2.7 ± 1.31%	-

* ++, excellent degradation effect, degradation efficiency > 50%; +, average degradation effect, 5% < degradation Efficiency < 50%; -, no degradation, degradation Efficiency < 5%. ** Degradation efficiency ranges from the lowest degradation efficiency to the highest degradation efficiency in parallel samples. *** +, the medium has a clear color change; -, the medium has no color change.

**Table 2:** Comparison of the degradation effect of strain TJFP1 with other reported phenanthrene-degrading bacteria.

Strain	Phenanthrene (mg/L)	Degradation Efficiency	Degradation Rate (mg/L/day)	Reference
TJFP1	100	100% (4.5 day)	24.48	This study
*Pseudomonas* sp. ANT13_1	50	22.4% (15 days)	0.75	[51]
*Pseudarthrobacter* sp. L1SW	500	96.3% (72 h)	160.50	[41]
*Klebsiella pneumoniae* SJK1	1250	40.5(12 days)	42.19	[52]
*Providencia rettgeri* VMP5	50	98.6% (4 days)	12.33	[53]
*Bacillus tropicus* VMP4	50	89.9% (4 days)	11.24	[53]
*Pseudarthrobacter phenanthrenivorans* A-5	50	79.54% (7 days)	5.68	[54]
*Stenotrophomonas* in *dicatrix* CPHE1	10	100% (21 days)	0.48	[37]
*Pseudomonas* sp. Lphe-2	100	92.76% (7 days)	13.25	[36]
*Pseudomonas* fluorescens AH-40	150	97% (15 days)	9.70	[55]
*Diaphorobacter* sp. Phe15	50	22% (15 days)	0.73	[56]
*Sphingobium* sp. AM	100	87.4% (7 days)	12.49	[57]
*Pseudomonas* sp. Ph6-gfp	50	81.1% (15 days)	2.70	[58]
*Massilia* sp. Pn2	150	95% (48 h)	71.25	[59]
*Mycolicibacterium* sp. A1-PYR	10	99% (2 days)	4.95	[60]
*Pseudomonas* sp. USTB-RU	100	86.6% (8 days)	10.83	[61]
*Pseudomonas* sp. Ph-3	100	90% (7 days)	12.86	[62]
*Roseovarius* sp. SBU1	100	28.4% (10 days)	2.84	[63]
*Pseudarthrobacter* sp. Sphe3	400	90% (4 days)	90.00	[64]
*Pseudarthrobacter* sp. J015	100	90% (4 days)	22.50	[65]
*Arthrobacter* sp. P1–1	40	99% (7 days)	5.66	[66]
*Arthrobacter* sp. YC-RL1	50	82.3% (5 days)	8.23	[67]
*Arthrobacter* sp. K3	250	99% (5 days)	49.50	[68]
*Mycolicibacterium*. WY10	100	100% (60 h)	40	[69]

**Table 3:** The number of protein-coding genes in different databases.

Databases	Number of Protein-Coding Genes	%
NR	6777	96.70
GO	1413	20.85
eggnog	5320	376.50
KEGG	2832	53.23
Swiss	3671	129.63
CAZy	217	5.91
CARD	106	48.85

**Table 4:** Alpha diversity index for different treatment groups.

Sample		Shannon	Simpson	ACE	Chao1	Pielou	Coverage
Treatment	Replicate						
NC	1	4.916584	0.015235	702.4928	704.6786	0.755206	0.999071
	2	4.885554	0.017757	728.6357	721.00	0.747903	0.998949
	3	4.836199	0.018395	753.4264	752.3582	0.737588	0.998766
PT	1	2.825297	0.173504	575.9003	577.1613	0.452611	0.998644
	2	3.095102	0.145655	589.6721	590.4355	0.491655	0.998812
	3	2.77461	0.18746	589.3641	591.381	0.443802	0.998537
NG-5DAY	1	3.467205	0.099935	553.1702	549.2542	0.555444	0.99901
	2	3.483609	0.100303	608.2021	616.1724	0.553046	0.998598
	3	3.549557	0.091821	571.5692	565.9242	0.564177	0.999101
NG-20DAY	1	3.19783	0.096232	528.1885	518.0845	0.518144	0.998857
	2	3.182169	0.10002	508.6596	502.3871	0.51901	0.998888
	3	3.24315	0.094227	496.7473	497.06	0.52821	0.999116
BG-5DAY	1	3.088348	0.142454	520.5665	514.25	0.501257	0.998933
	2	3.09602	0.125491	565.0113	561.0909	0.497072	0.998705
	3	2.809184	0.169538	515.8918	525.1429	0.45916	0.99872
BG-20DAY	1	3.123404	0.111245	515.0163	509.35	0.509788	0.998796
	2	3.045859	0.106694	497.9351	489.5	0.497487	0.998979
	3	3.093912	0.111542	491.1816	497.1633	0.507359	0.998903

The table presents the following diversity metrics: Shannon index and Simpson index reflect community diversity; ACE and Chao1 estimators reflect species richness; Pielou’s evenness index reflects community evenness; Coverage reflects sequencing depth. Abbreviations: NC, no treatment Control Group (uncontaminated original soil); PT, pollution Treatment Group (contaminated control); NG-5DAY, natural degradation for 5 days (no inoculum); NG-20DAY, natural degradation for 20 days (no inoculum); BG-5DAY, bioremediation for 5 days (inoculated with the *Mycobacterium* sp., TJFP1); BG-20DAY, bioremediation for 20 days (inoculated with the *Mycobacterium* sp., TJFP1).

## Data Availability

The whole genome sequencing data generated in this study are available in the NCBI GenBank database under CP187453-CP187455. The raw soil microbial diversity data generated in this study have been deposited in the NCBI Sequence Read Archive (SRA) under BioProject accession PRJNA1242019. All data are publicly accessible without restrictions.

## Appendix A

**Table A1:** Annotated PAH-related degradation genes.

Gene ID	Gene	Position	Length	Predicted Function	Identity
TJFP1000810	phtAa	820437–821900	1463	phthalate 3,4-dioxygenase, alpha subunit	99%
TJFP1000811	phtAb	821945–822496	551	phthalate dioxygenase small subunit	98.9%
TJFP1000813	phtB	822795–823598	803	phthalate 3,4-cis-dihydrodiol dehydrogenase	100%
TJFP1000814	phtAc	823627–823827	200	phthalate 3,4-dioxygenase ferredoxin component	98.5%
TJFP1000815	phtAd	823824–825065	1241	phthalate 3,4-dioxygenase ferredoxin reductase subunit	97.8%
TJFP1000816	phdI	825643–826728	1085	1-hydroxy-2-naphthoate dioxygenase	98.6%
TJFP1000817	phdJ	826747–827751	1004	4-(2-carboxyphenyl)-2-oxobut-3-enoate aldolase	99.7%
TJFP1000818	phdF	827770–828660	890	putative 2,3-dihydroxybiphenyl 1,2-dioxygenase	99.7%
TJFP1000825	nidB	832716–833225	509	aromatic-ring-hydroxylating dioxygenase subunit beta	100%
TJFP1000828	nidD	835603–837063	1460	aldehyde dehydrogenase	100%
TJFP1000833	nidB	840539–841084	545	aromatic-ring-hydroxylating dioxygenase subunit beta	100%
TJFP1000834	nidA	841145–842512	1367	aromatic-ring-hydroxylating dioxygenase subunit alpha	100%
TJFP1000853	phdK	860418–861791	1373	2-formylbenzoate dehydrogenase	100%
TJFP1000856	nidA	864270–865661	1391	aromatic-ring-hydroxylating dioxygenase subunit alpha	100%
TJFP1000857	nidB	865658–866200	542	aromatic-ring-hydroxylating dioxygenase subunit beta	100%
TJFP1000872	pht4	879197–880426	1229	phthalate 4,5-cis-dihydrodiol dehydrogenase	99.3%
TJFP1000877	phdF	883813–884703	890	putative 2,3-dihydroxybiphenyl 1,2-dioxygenase	99.3%
TJFP1000878	nidA	884776–886176	1400	aromatic-ring-hydroxylating dioxygenase subunit alpha	99.6%
TJFP1000879	nidB	886241–886759	518	aromatic-ring-hydroxylating dioxygenase subunit beta	100%
TJFP1000892	pcaH	898863–899726	863	protocatechuate 3,4-dioxygenase subunit beta	99%
TJFP1000893	pcaG	899726–900295	569	protocatechuate 3,4-dioxygenase subunit alpha	97.9%

**Table A2:** Differences in relative abundance at the gate level between treatment groups (relative abundance > 1%).

Sample Name	NC	PT	NG-5DAY	NG-20DAY	BG-5DAY	BG-20DAY
Firmicutes	14.42 ± 2.21% ^abc^	60.32 ± 2.96% ^a^	51.16 ± 2.21% ^abc^	45.58 ± 3% ^c^	57.06 ± 10.58% ^ab^	48.72 ± 1.2% ^bc^
Actinobacteria	19.63 ± 1.89% ^bc^	25.36 ± 1.11% ^cd^	32.16 ± 1.89% ^bc^	44.11 ± 2.72% ^a^	31.49 ± 9.79% ^c^	41.45 ± 2.74% ^ab^
Proteobacteria	19.71 ± 0.38% ^b^	4.54 ± 0.34% ^c^	7.21 ± 0.38% ^b^	4.18 ± 0.49% ^c^	6.40 ± 0.22% ^b^	4.06 ± 0.6% ^c^
Chloroflexi	15.71 ± 0.47% ^b^	3.62 ± 0.49% ^b^	3.56 ± 0.47% ^b^	1.67 ± 0.16% ^c^	1.75 ± 0.57% ^c^	1.25 ± 0.21% ^c^
Acidobacteriota	13.95 ± 0.54% ^b^	2.48 ± 0.72% ^b^	1.77 ± 0.54% ^b^	0.82 ± 0.13% ^b^	0.81 ± 0.37% ^b^	0.68 ± 0.32% ^b^
Gemmatimonadota	5.42 ± 0.29% ^b^	1.07 ± 0.23% ^b^	1.29 ± 0.29% ^b^	1.13 ± 0.17% ^b^	0.81 ± 0.06% ^b^	1.17 ± 0.46% ^b^
Myxococcata	1.90 ± 0.06% ^b^	0.30 ± 0.01% ^c^	0.70 ± 0.06% ^b^	0.44 ± 0.09% ^c^	0.43 ± 0.07% ^c^	0.38 ± 0.04% ^c^
Patescibacteria	0.69 ± 0.21% ^a^	0.20 ± 0.07% ^a^	0.44 ± 0.21% ^a^	0.82 ± 0.29% ^a^	0.25 ± 0.06% ^a^	1.03 ± 0.63% ^a^
Bacteroidota	1.74 ± 0.02% ^b^	0.28 ± 0.05% ^b^	0.16 ± 0.02% ^b^	0.51 ± 0.25% ^b^	0.09 ± 0.01% ^b^	0.57 ± 0.44% ^b^
Methylxorhablous	1.25 ± 0.02% ^b^	0.27 ± 0.04% ^b^	0.24 ± 0.02% ^b^	0.15 ± 0.04% ^c^	0.14 ± 0.02% ^c^	0.12 ± 0.02% ^c^
Others	5.76 ± 0.25% ^bc^	1.55 ± 0.37% ^b^	1.34 ± 0.25% ^bc^	0.63 ± 0.04% ^cd^	0.78 ± 0.14% ^cd^	0.58 ± 0.09% ^d^

Different letters in the same row indicate significant differences (*p* < 0.05). Data are mean ± standard deviation (n = 3).

**Table A3:** Differences in relative abundance at the genus level across treatment groups (top 20 in abundance).

Genus	NC	PT	NG-5DAY	NG-20DAY	BG-5DAY	BG-20DAY
*Bacillus*	5.34 ± 1.92% ^d^	33.42 ± 2.07% ^a^	24.73 ± 1.13% ^bc^	20.66 ± 1.79% ^c^	30.71 ± 5.95% ^ab^	24.99 ± 1.95% ^bc^
*Fictibacillus*	0.11 ± 0.04% ^e^	20.83 ± 1.15% ^a^	6.9 ± 0.82% ^cd^	6.37 ± 0.49% ^d^	9.85 ± 1.01% ^b^	8.3 ± 0.82% ^bc^
*Pseudonocardia*	0.12 ± 0.04% ^d^	2.45 ± 0.14% ^c^	15.3 ± 1.3% ^b^	17.1 ± 1.37% ^a^	1.11 ± 0.48% ^cd^	2.13 ± 0.46% ^c^
*Mycobacterium*	0.33 ± 0.11% ^b^	0.15 ± 0.01% ^b^	0.11 ± 0.04% ^b^	0.06 ± 0.02% ^b^	16.42 ± 5.69% ^a^	14.07 ± 2.4% ^a^
*Marmoricola*	0.83 ± 0.17% ^b^	0.44 ± 0.03% ^b^	1.97 ± 0.6% ^b^	8.53 ± 1.43% ^a^	1.77 ± 0.64% ^b^	9.74 ± 1.89% ^a^
*Streptomyces*	1.1 ± 0.15% ^c^	6.16 ± 0.23% ^a^	2.86 ± 0.5% ^bc^	2.55 ± 0.14% ^bc^	3.89 ± 2.44% ^ab^	2.46 ± 0.2% ^bc^
*Saccharomonospora*	0.04 ± 0.01% ^d^	8.62 ± 1.02% ^a^	1.08 ± 0.25% ^bc^	1.56 ± 0.61% ^b^	0.73 ± 0.25% ^bcd^	0.52 ± 0.1% ^cd^
*Micromonospora*	0.33 ± 0.08% ^a^	0.46 ± 0.1% ^a^	2.21 ± 0.14% ^b^	2.59 ± 0.13% ^b^	2.49 ± 0.53% ^b^	3.38 ± 0.24% ^a^
*Nocardioides*	0.41 ± 0.1% ^b^	0.19 ± 0.02% ^b^	0.75 ± 0.25% ^b^	4.71 ± 1.11% ^a^	0.65 ± 0.26% ^b^	4.24 ± 0.24% ^a^
*Paenibacillus*	0.57 ± 0.1% ^b^	0.4 ± 0.04% ^b^	2.05 ± 0.77% ^a^	2.17 ± 0.42% ^a^	2.11 ± 0.19% ^a^	2.17 ± 0.33% ^a^
*norank_f__JG30-KF-CM45*	2.66 ± 0.61% ^a^	0.88 ± 0.05% ^c^	1.77 ± 0.7% ^b^	0.81 ± 0.13% ^c^	0.88 ± 0.21% ^c^	0.68 ± 0.05% ^c^
*Paenisporosarcina*	0.46 ± 0.2% ^b^	0.35 ± 0.06% ^b^	2.96 ± 0.71% ^a^	0.18 ± 0.03% ^b^	3.48 ± 2.31% ^a^	0.18 ± 0.03% ^b^
*norank_o__Vicinamibacterales*	4.76 ± 1.23% ^a^	0.93 ± 0.34% ^b^	0.66 ± 0.17% ^b^	0.33 ± 0.06% ^b^	0.32 ± 0.16% ^b^	0.27 ± 0.18% ^b^
*Sphingomonas*	2.84 ± 1.09% ^a^	0.60 ± 0.04% ^b^	1.36 ± 0.15% ^b^	0.57 ± 0.06% ^b^	0.92 ± 0.04% ^b^	0.47 ± 0.06% ^b^
*Arthrobacter*	2.67 ± 0.95% ^a^	0.55 ± 0.02% ^b^	1.16 ± 0.1% ^b^	0.79 ± 0.06% ^b^	0.66 ± 0.22% ^b^	0.65 ± 0.02% ^b^
*Kroppenstedtia*	1.49 ± 0.92% ^a^	0.28 ± 0.03% ^a^	0.7 ± 0.09% ^a^	0.89 ± 0.42% ^a^	1.86 ± 1.92% ^a^	0.53 ± 0.07% ^a^
*norank_c__MB-A2-108*	3.34 ± 0.7% ^a^	1.23 ± 0.35% ^b^	0.49 ± 0.15% ^c^	0.23 ± 0.02% ^c^	0.22 ± 0.11% ^c^	0.15 ± 0.07% ^c^
*norank_f__Gemmatimonadaceae*	3.22 ± 0.56% ^a^	0.66 ± 0.11% ^b^	0.66 ± 0.09% ^b^	0.31 ± 0.06% ^b^	0.43 ± 0.03% ^b^	0.24 ± 0.02% ^b^
*norank_f___Vicinamibacteraceae*	3.81 ± 0.86% ^a^	0.64 ± 0.19% ^b^	0.43 ± 0.13% ^b^	0.19 ± 0.03% ^b^	0.18 ± 0.09% ^b^	0.16 ± 0.08% ^b^
*Rhodococcus*	0.47 ± 0.52% ^bc^	0.03 ± 0.00% ^c^	1.26 ± 0.22% ^b^	2.41 ± 0.46% ^a^	0.14 ± 0.03% ^c^	0.75 ± 0.69% ^bc^

Different letters in the same row indicate significant differences (*p* < 0.05). Data are mean ± standard deviation (n = 3).
